# Boronate Derivatives of Functionally Diverse Catechols: Stability Studies

**DOI:** 10.3390/molecules15042347

**Published:** 2010-03-31

**Authors:** Kamal Aziz Ketuly, A. Hamid A. Hadi

**Affiliations:** Chemistry Department, Faculty of Science, University Malaya, 50603 Kuala Lumpur, Malaysia; E-Mail: ahamid@um.edu.my (A.H.A.H.)

**Keywords:** cyclic benzeneboronate, catechols, boronic acids

## Abstract

Benzeneboronate of catecholic carboxyl methyl esters, N-acetyldopamine, coumarin and catechol estrogens were prepared as crystalline derivatives in high yield. Related catechol compounds with extra polar functional group(s) (OH, NH_2_) do not form or only partially form unstable cyclic boronate derivatives.

## 1. Introduction

The selective reactions of boronic acids with compounds containing two or more suitably disposed proximal polar functional groups (OH, NH, SH, COOH) can afford cyclic derivatives of reduced polarity, of value in analytical characterization and in separation of polyfunctional compounds [1,2]. Crystalline benzeneboronates from the reactions of benzeneboronic acid with polyols [3], sugars [4,5], other carbohydrates [6,7,8], macrolide aglycones [9] and steroids [10] have been isolated and characterized. Depending on the substrates, the condensations could take place in aqueous and aqueous-methanol solutions [3], organic solventc [6], reactions by fusion techniques, or by reaction in anhydrous solvents followed by azeotropic removal of water [5]. Boronate derivatives have a wide range of applications in: separation of isomeric pairs of *cis* and *trans* diols [11] and as extracting probes to capture and enrich *cis*-diol-containing biomolecules [12]; The differences in the solubility of the boronates of isomeric carbohydrates greatly facilitate their separation by fractional re-crystallization [13]; Boronate affinity chromatography (BAC) is an important tool for specific capture and separation of *cis*-diol-containing compounds such as glycoproteins, RNA and carbohydrates [14]. Systematical investigation on the retention mechanism revealed that multiple intermolecular interactions occur between the analytes and the boronate affinity monolith, including boronate affinity, reversed-phase, cation-exchange and hydrogen bonding interactions, depending on the conditions used [15]; A variety of cyclic boronates that are stable to hydrolysis has been studied by reversed-phase high pressure liquid chromatography (HPLC) [16]. Gas chromatography-mass spectroscopy (GC-MS), of cyclic boronates is valuable and thus relatively highly volatile derivatives of a great variety of the less volatile polar multifunctional compounds, including many of biological importance [17,18,19,20], may be analyzed. Structural information which can be acquired for specific ions, associated with the MS fragmentation of the new functionality, the identification of these, and of other ions containing boron was facilitated by the characteristic isotope ratio of boron (B^11^:B^10^, 4:1) [21]; Aryl and heteroaryl boronates are very important intermediates in organic synthesis that have been deployed in many useful transformations [22,23,24] and for the determination of absolute configuration [25]. The rigidity of the cyclic boronate derivatives of corresponding 1,2- or 1,3-diols, requiring relatively fixed conformations, is used in nuclear magnetic resonance (NMR) studies of carbohydrate concentrations and the position of their functional groups [26]. The B^11^-NMR resonance of boron esters is a broad line and its spectra allow distinction between five, six, seven-member cyclic and acyclic boronates [27,28,29]. Suitable boronate single crystals are useful for X-ray diffraction to establish the conformation and the absolute configuration of the molecule in the crystalline state [22,30,31]. Boronates are used as catalysts, in the complexes of areneboronic acids with carbohydrates, formed in aqueous solution in a manner similar to that of borate [32]. The use of boron-containing compounds in the treatment of cancer has revolved about the unique nuclear property of the non-radioactive B^10^ isotope with a high cross section to absorb thermal neutrons with the liberation of much energy selectively destroying or weakening cancerous cells [33].

**Scheme 1:**
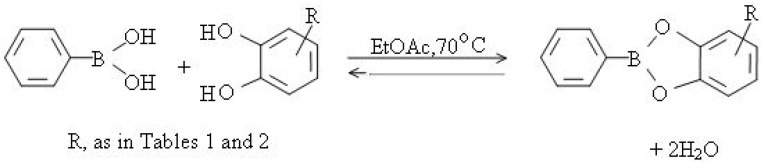
Typical reaction of benzeneboronic acid with catechols.

In the present work we focused on the formation and stabilities of cyclic boronate esters of various types of biologically important catechols. Several crystalline cyclic boronate esters of catechols: 3,4-dihydroxy methyl esters **1**–**4**; N-acetyldopamine (**5**), estra-1,3,5(10)-triene-3,4-diol (**6**) and 4-methyl-7,8-dihydroxycoumarin (**7**) were obtained (Table 1), Scheme 1.

**Table 1:** Preparative benzeneboronate derivative formation from catechol type compounds.

Parent compound	Compound No	Amount used mg (mmol)	Benzeneboronic acidmg (mmol)	Data corresponding benzeneboronates					
				Amount recovered mg ^a^(% crude yield)	m.p. °C	Mol Formula (Mol. Wt)	Analysis		MS
							Found	Calc.	M^+ ^(%) ^b^
Methyl 3,4-dihydroxybenzoate ^**c**^	1	831	629.5	1050		C_14_H_11_BO_4_	C, 65.90	C, 66.14	254
		(4.50)	(5.15)	(84)	109.5-110.5 **^ d^**	(254)	H, 4.23	H, 4.33	(100)
							B, 4.78	B, 4.3	
Methyl -3,4-dihydroxy-phenylacetate	2	300	203	400		C_15_H_11_BO_4_	C, 67.20	C, 67.16	268
		(1.65)	(1.67)	(90.4)	82-83 **^e^**	(268)	H, 4.95	H, 4.85	(97)
							B, 3.8	B, 4.1	
Methyl 3,4-dihydroxydihydro-cinnamate	3	500	317	680		C_16_H_15_BO_4_	C, 68.24	C, 68.09	282
		(2.55)	(2.6)	(95)	61-62 **^f^**	(282)	H, 5.38	H, 5.32	(100)
							B, 3.83	B, 3.9	
Methyl 3,4-dihydroxycinnamate	4	263	170	349		C_16_H_13_BO_4_	C, 68.7	C, 68.57	280
		(1.36)	(1.39)	(92)	131-133 **^g^**	(280)	H, 4.35	H, 4.64	(100)
							B, 4.01	B, 4.0	
*N*-Acetyldopamine	5	977	612	1405		C_16_H_16_BNO_3_	C, 68.0	C, 68.33	281
		(5.01)	(5.02)	(99.8)	159-160 **^h^**	(281)	H, 5.90	H, 5.69	(6)
							B, 4.11	B, 3.90	
							N, 5.06	N, 4.98	
Estra-1,3,5(10)-triene-3,4-diol **^i^**	6	22	10	28	176-177 **^j^**	C_24_H_27_BO_2_	C, 80.42	C, 80.45 ^**j**^	358
		(0.08)	(0.08)	(96)		(358)	H, 7.34	H, 7.54	(100)
4-Methyl-7,8-dihydroxy-coumarin ^**k**^	7	260	165	370	191-193 **^l^**	C_16_H_11_BO_4_	**l**		278
		(1.35)	(1.35)	(98.4)		(278)			(100)

Notes:

- ^a ^GLC analysis (GC column as in b) of these crude products showed in each case a single peak corresponding to their benzeneboronate derivatives. Traces of excess benzeneboronic acid (eluted as triphenylboroxine) were also observed.
- ^b ^Mass spectral data were recorded at electron energy 20 eV, using an LKB 9000 GC-MS instrument, fitted with a glass column (2 m × 4 mm, i.d.) of 1% OV-1 on Gas Chrom Q (100–120 mesh). The flash heater was at 250 °C, the molecular separator at 270 °C, and ion source at 265 °C. The helium carrier gas flow rate was 30 mL/min. The trap current was 60 mA, filament current 4 A and accelerating voltage 3.5 KV. In Table 1 the abundances for the ion are shown in brackets ( ).
- ^c ^The isomeric methyl 2,3-dihydroxybenzoate did not react fully with benzeneboronic acid, as judged by GLC. On recrystallisation of the crude reaction mixture, from acetone-hexane; the recovered material was largely benzeneboronic acid. Vacuum sublimation of the crude product, also failed to yield any cyclic ester.
- ^d ^Recrystallisation from acetone-hexane; m.p. 107–108.5 °C; then vacuum sublimation: yield a middle fraction which was collected.
- ^e ^Vacuum sublimation at 55 °C/0.01 torr.
- ^f ^Vacuum sublimation at 55 °C/0.01 torr, yielded fine white crystals.
- ^g ^Recrystallisation from acetone-hexane; m.p. 125–127 °C; then followed by vacuum sublimation; a middle fraction was collected.
- ^h ^Vacuum sublimation at 130 °C/torr; m.p. 157–160 °C, a second sublimation yielded fine white crystals.
- ^i ^The isomeric 2-hydroxy-17-deoxoestrone benzeneboronate vacuum sublimation at 140 °C/0.01 torr; yielded a viscous gum, which failed to crystallize from acetone, EtOAc or hexane. Trituration in hexane at low temperature yielded a semi-solid product, which crystallized on standing m.p. 244–247 °C. GC showed traces of benzeneboronic acid. GC-MS, M^+^358 (100%), a satisfactory micro-analysis or HRMS was not adequate for this compound. This is due to partial hydrolysis on storage. The ^1^H-NMR showed satisfactory results.
- ^j ^Recrystallisation from acetone; then vacuum sublimation at 160 °C/0.01 torr. The sublimed material was recrystallised twice from acetone. High resolution MS, C_24_H_27_BO_2_, requires 358.2810; found 358.2104.
- ^k ^The isomeric 4-methyl-6,7-dihydroxycoumarin benzeneboronate was formed and showed GC-MS peak with M^+ ^278 (100%) but attempts to isolate pure crystals were unsuccessful.
- ^l ^Two recrystallisation from CHCl_3_-hexane, then vacuum sublimation at 140 °C/0.01 torr, gave a middle fraction which was recrystallised from EtOAc-hexane. GLC analysis for this product showed the presence of ca. 0.1% of benzeneboronic acid: the micro-analysis was not satisfactory for carbon. High resolution MS, C_16_H_11_BO_4_, requires 278.0672; found 278.0750.

**Figure 2:**
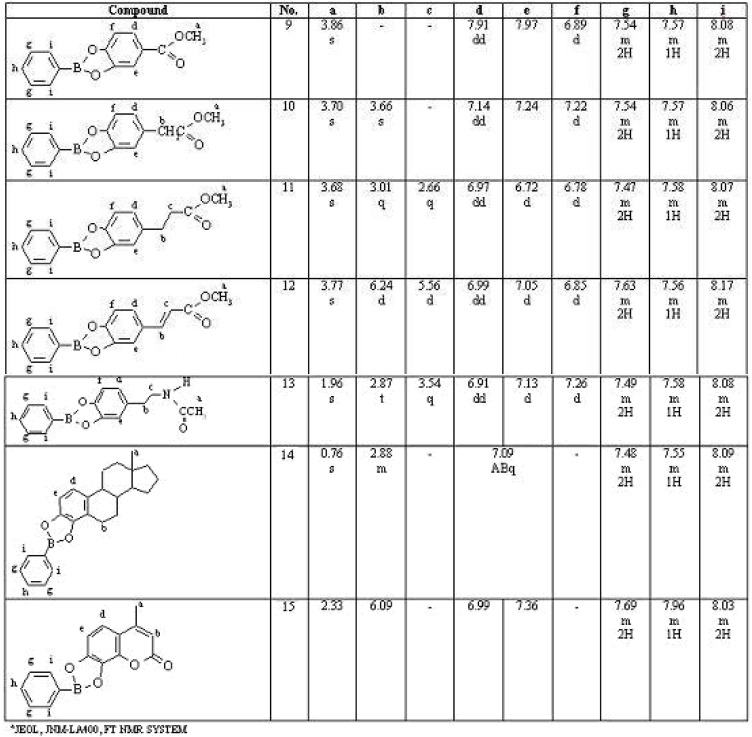
^1^H-NMR, characteristic proton chemical shifts (=ppm), of the catechol-type benzeneboronate derivatives. Data measured in CDCl_3_ + 0.4% tetramethylsilane, (400 MHz). ^*^

## 2. Results and Discussion

The compounds in Figure 2, readily prepared under neutral conditions in EtOAc at 70 °C, resulted from the spontaneous reactions of 0.1 molar excess of benzeboronic acid with the catechol group compounds **1**–**7 **in (Table 1).

The recovered crude yields of the cyclic boronates were high (84–99.8%). The esters were stable in anhydrous non-hydroxylic organic solvents (e.g., EtOAc, pyridine, hexane, toluene) and towards aerial oxidation and atmospheric moisture to some extent and remained stable under storage. However, they were unstable on thin-layer chromatography (TLC) or on 'dry column' silica gel chromatography, undergoing disassociation with subsequent partial oxidation. This resulted in hydrolysis of the boronates to their starting materials (the catechol + boronic acid) followed by the aerial oxidation of the catechols to their quinine forms.

The gas chromatography-mass spectrometry (GC-MS) of the benezeneboronates of compounds **1–7** were studied. These results (Table 1) showed that the molecular ions are the base peaks for all the boronate esters, except for compounds (**2,** 97%) and (**5**, 6%), [34]. Many of the principal ions in the mass spectra retain boron in their structure. The high resolution mass spectral (HRMS) direct probe for the benzeneboronates of compounds **6** and **7**, were measured to confirm their molecular formula (Table 1). The mass spectral fragmentation pattern of the isomeric pairs: 4-methyl-6,7- (and -7,8-)-dihydroxycoumarin and 2- and 4-hydroxy-17-deoxoestrone (estra-1,3,5(10)-triene-3,4-diol), showed no significant differences; but GC succeeded in separating these isomers. In our GC and GC-MS studies of the cyclic boronates, of all the catecholic compounds mentioned in this paper, it has been established that their ease of displacement by acetylating or trimethylsilylating (TMS) reagents allows the application of sequential derivatization procedures. These are the useful features for the analysis of biologically important catechols, which usually exist in low concentrations. 

The Infrared spectra of the cyclic boronate derivatives of compounds **1**–**7** exhibited two characteristic bands, for B-O and Ph-O absorption in the regions 1,325-1,380 cm^−1^ and 1,390-1,455 cm^−1^, respectively. The other bands were characteristic for the remaining functional groups of the listed compounds [5,8,35].

The cyclic boronate derivatives of the compounds listed in Figure 2 showed characteristic ^1^H-NMR chemical shifts for the five aromatic protons of the phenyl group bonded directly to the boron atoms [36,37,38]. The two protons *ortho* to the boron atom are deshielded, and resonated at δ 8.06–8.87; the two *meta* protons resonated at higher field, in the region δ 7.47–7.69; whilst the single *para* proton resonated at δ. 6.55–7.96. The corresponding protons in the catechol boronate unit appeared in the δ 6.72–7.97 region [38,39,40,41], as shown in Figure 2. 

The compounds methyl 2,3-dihydroxybenzoate; 2,3-dihydroxybenzaldehyde; 2,3,4-trihydroxy-acetophenone; dopamine; 3-(3,4-dihydroxyphenyl)-L-alanine methyl ester; α-propyldopacetamide; 3,4-dihydroxy-(isopropylamino)acetophenone; salsolinol (1-methyl-6,7-dihydroxy-1,2,3,4-tetrahydro-isoquinoline); 3,4-dihydroxynomifensine; apomorphine; *N*-*n*-propylnorapomorphine and 2-hydroxy-estradiol; all formed the cyclic boronate esters, as judged by GC-MS, but these products were not stable and could not be isolated or purified, due to hydrolysis followed by aerial oxidation at ambient temperature. 3,4-Dihydroxy-α-hydroxyphenylacetic acid, tetrahydroxyterephthalic acid diethyl ester, brazilin and gossypol did not yield cyclic boronate derivatives either on an analytical or preparative scale. For some of these compounds, the failure to form stable cyclic boronates may reflect either additional polar functionality or may be due to a reduction in reactivity arising from the strong inter-molecular hydrogen bonding to the catechol hydroxyl groups. Dopamine-3,4-benzeneboronate proved to be highly unstable in air and decomposed to a dark oil. This compound when acetylated yields dopamine triacetate and not compound **13**. The benzeneboronate of the isomeric pairs: 2-hydroxy-17-deoxoestrone and 4-methyl-6,7-dihydroxycoumarin were more susceptible to partial hydrolysis on storage than their isomers **14 **and **15 **respectively**. **The benzeneboronates of the compounds in (Table 1) are fine crystals and were not suitable for X-ray diffraction structural analysis. 

## 3. Experimental

### General

The reagents were obtained from British Drugs House (BDH), except benzeneboronic acid which was from Koch-Light Labs, Ltd. The substrates were obtained from Fluka, BDH, Sigma Chemicals Co., Aldrich Chem. Co., Koch-Light Labs, Ltd. and Makor and the rest were a gift from Professor C.J.W. Brooks (Chemistry Department - Glasgow University). The Infrared (IR) spectra were measured on a Perkin-Elmer Grating Infra Red Spectrophotometer Model 257. The Nuclear Magnetic Resonance (NMR) instrument used was JEOL JNM-LA400, FT NMR System. Spectra were recorded in CDCl_3_ containing 0.4% tetramethylsilane as internal standard. The mass spectra (direct insertion probe) were measured on a VG MICROMASS 2S8 instrument. Melting points (m.p.) were recorded on a Kofler block.

*Methyl esters of acids containing catechol groups***1**–**4**: The substrates were refluxed in methanolic HCl [prepared by cautious addition of acetyl chloride (10 mL) to dry methanol (60 mL)] for 4 h, monitored by TLC, and the products were recovered in the normal way. 

*N-Acetyldopamine* (**5**): This compound was prepared from dopamine by a modification of the procedure of [40]. Dopamine hydrobromide (2.051 g, 8.8 mmol) was dissolved in water (4 mL) and ethyl acetate (20 mL) was added to this solution. A solution of acetic anhydride (0.895 mL, 9.5 mmol) in methanol (20 mL) was added at room temperature. The crude product (1.670 g, 97.7%) was recovered with high yield. This was purified firstly through a short column silica gel and then by vacuum sublimation and a colorless gummy oil was recovered (MS, M^+^ 195). This product could not be crystallized from different solvent systems and it was sensitive to aerial oxidation. The corresponding benzeneboronate is crystalline, m.p. 159–160 °C (Table 1).

*3,4-Dihydroxyestra-1,3,5(10)-triene* (**6**): Firstly, 3-hydroxyestra-1,3,5(10)-triene was prepared from estrone according to a procedure by [42] and this was followed by the procedure of [43] to obtain the isomers: 2,3- and 3,4-dihydroxyestra-1,3,5(10)-triene.

*Typical procedure for the preparation of benzeneboronate derivatives: Methyl 3,4-dihydroxy-dihydrocinnamate 3,4-benzeneboronate* (**11**): Methyl 3,4-dihydroxydihydrocinnamate (**3**, 0.5 g, 2.5 mmol) was dissolved in EtOAc (5 mL); benzeneboronic acid (0.317 g, 2.6 mmol) in EtOAc (2 mL) was added, and the reaction mixture heated for 20 min at 70 °C. After removal of the solvent, the gummy resulting product was dissolved in ether. Evaporation of the solvent with nitrogen yielded a crystalline material (680 mg, 95%), m.p. 60–62 °C. GLC showed a single well-defined peak. Vacuum sublimation yielded fine white crystals, m.p. 61–62 °C. For analytical data and mass spectrometer (MS) are as in Table 1.

## 4. Conclusions

Stable cyclic benzeneboronate esters of biologically important catechols were formed on a micro-analytical scale and in preparative scale as crystalline products. Some of the catechols with extra polar functional group(s) (OH, NH_2_) did form cyclic boronates but these were not stable, while others failed to form any cyclic derivatives.
